# Effect of Hepatocellular Carcinoma Surveillance Programmes on Overall Survival in a Mixed Cirrhotic UK Population: A Prospective, Longitudinal Cohort Study

**DOI:** 10.3390/jcm10132770

**Published:** 2021-06-24

**Authors:** Mohammad Inamul Haq, Thomas M. Drake, Tee Lin Goh, Asma Ahmed, Ewan Forrest, Stephen Barclay, Ruth Gillespie, Mathew Priest, Jeff Evans, Janet Graham, Stuart Ballantyne, Donald C. McMillan, Peter C. Hayes, Thomas G. Bird, Adrian J. Stanley

**Affiliations:** 1 Department of Gastroenterology, Glasgow Royal Infirmary, Glasgow G4 0SF, UK; Ewan.Forrest@ggc.scot.nhs.uk (E.F.); stephenbarclay@nhs.net (S.B.); Ruth.Gillespie@ggc.scot.nhs.uk (R.G.); Adrian.stanley@ggc.scot.nhs.uk (A.J.S.); 2 Department of Clinical Surgery, 51 Little France Crescent, University of Edinburgh, Edinburgh EH16 4SA, UK; t.drake@ed.ac.uk (T.M.D.); dcmm1w@clinmed.gla.ac.uk (D.C.M.); 3 Institute of Cancer Sciences College of Medical, Veterinary and Life Sciences, University of Glasgow, Glasgow G61 1BD, UK; Jeff.Evans@ggc.scot.nhs.uk (J.E.); t.bird@beatson.gla.ac.uk (T.G.B.); 4 Centre for Medical Informatics, Usher Institute, University of Edinburgh, Edinburgh EH16 4UX, UK; 5 Department of Medicine, Dumfries and Galloway Royal Infirmary, Dumfries DG2 8RX, UK; TeeLin.Goh@nhs.scot; 6 Department of Gastroenterology, Forth-Valley Royal Hospital, Larbert FK5 4WR, UK; asma.ahmed@nhs.scot; 7 Department of Gastroenterology, Gartnavel General Hospital, Glasgow G12 0YN, UK; Mathew.Preist@ggc.scot.nhs.uk; 8 Department of Oncology, Beatson West of Scotland Cancer Centre, Glasgow G12 0YN, UK; Janet.Graham@ggc.scot.nhs.uk; 9 Department of Radiology, Queen Elizabeth University Hospital, Glasgow G51 4TF, UK; Stuart.Ballantyne@ggc.scot.nhs.uk; 10 Centre for Liver and Digestive Disorders, Royal Infirmary of Edinburgh, Edinburgh EH16 4SA, UK; p.hayes@ed.ac.uk; 11 Centre for Inflammation Research, The Queen’s Medical Research Institute, University of Edinburgh, Edinburgh EH1 64TJ, UK; 12 Cancer Research UK Beatson Institute, Glasgow G61 1BD, UK

**Keywords:** HCC, hepatocellular carcinoma, cirrhosis, surveillance, cancer screening, lead-time

## Abstract

Introduction: Surveillance for hepatocellular carcinoma (HCC) is recommended by national and international guidelines. However, there are no trial data on whether surveillance improves clinical outcomes in a UK cirrhosis population of mixed aetiology. Our aim was to determine the impact of, and adherence to, surveillance on overall survival. Methods: We prospectively collected data on consecutive patients diagnosed with HCC between January 2009 and December 2015 at two large UK centres. We assessed outcomes depending on whether they had been entered into an HCC surveillance programme, and if they had adhered to that. Results: Out of 985 patients diagnosed with HCC in this study, 40.0% had been enrolled in a surveillance programme. Of these, 76.6% were adherent with surveillance and 24.4% were not. Adherence to surveillance was significantly associated with improved overall survival, even when accounting for lead-time bias using different approaches (HR for 270 days lead-time adjustment 0.64, 0.53 to 0.76, *p* < 0.001). Conclusions: When adjusted for lead-time bias, HCC surveillance is associated with improved overall survival; however, the beneficial effect of surveillance on survival was lower than reported in studies that did not account fully for lead-time bias.

## 1. Introduction

Hepatocellular carcinoma (HCC) is the most common primary liver cancer and remains a major public health burden worldwide [1,2]. Major increases have been reported in HCC incidence in the last 25 years, particularly in Europe and North America [3]. In the UK, there has been a 63% increase in incidence and 55% increase in mortality from HCC over the past decade [4,5], which is highest in Scotland [6]. Incidence rates for HCC are projected to rise by 38% (43% for males and 21% for females) in the UK by 2035, accompanied by a 58% rise in mortality [4].

Cirrhosis is the main risk factor for HCC, with up to a third of patients developing HCC. Patients with cirrhosis have a 1 to 5.8% risk per year of developing HCC [7], but regular surveillance may allow early detection and increase access to potentially curative therapies. Five-year survival rates for early stage HCC is more than 70%, compared with less than 5% when diagnosed at an advanced stage [8,9,10]. Most guidelines recommend surveillance is undertaken by 6-monthly ultrasound (US) scan, which has a sensitivity of 58–89% and specificity greater than 90%, and some centres also incorporate alpha-fetoprotein (AFP) measurement [11,12,13,14,15].

Lead-time bias greatly affects studies of interventions which aim to detect cancer early. When this bias arises, survival time is inflated, making an intervention appear to have a greater effect than it actually has [16]. Many non-randomised, observational studies have suggested a survival benefit from HCC surveillance [17,18], but did not investigate whether adherence to surveillance is associated with survival benefit. The aims of this study were to estimate the survival of patients diagnosed with HCC who had been entered into a surveillance programme, and to assess the effect of adherence to the programme on outcomes.

## 2. Materials and Methods

### 2.1. Study Design and Population

Consecutive patients who were diagnosed with HCC in a 7-year period from January 2009 to December 2015 were entered prospectively into this study. These patients were identified through regional multidisciplinary team meetings (MDTs) in Glasgow and Edinburgh, the two largest centres in Scotland. There are higher levels of socioeconomic deprivation in Glasgow than Edinburgh and we took this into account as described in our modelling process. Results are reported according to Strengthening the Reporting of Observational Studies in Epidemiology (STROBE) and Statistical Analyses and Methods in the Published Literature (SAMPL) guidelines [19,20]. To establish the denominator of patients who may have been eligible for inclusion in our study, we extracted the total number of patients with the ICD code ‘C22.0′ for Hepatocellular Carcinoma from the Scottish Cancer Registry.

### 2.2. Inclusion Criteria

The decision to enter a patient with known cirrhosis into a surveillance programme was made by the responsible Gastroenterologist or regional Hepatologist. The aetiology of underlying liver disease was determined by the patient’s history (including alcohol intake), clinical evaluation, laboratory parameters including viral and autoimmune serology, genetic testing where appropriate, imaging, and histology when available. The Barcelona Clinic Liver Cancer (BCLC) staging system was used to evaluate the HCC stage at diagnosis [21].

### 2.3. Outcomes

The primary outcome was overall survival, measured in years from diagnosis of HCC. Surviving patients were followed up until 1 August 2019.

### 2.4. Surveillance Adherence

A pragmatic approach for adherence with surveillance was adopted, with adherence defined as a US scan performed within a surveillance programme up to 9 months prior to HCC diagnosis. Based upon enrolment in the surveillance programme and adherence, patients were considered as never entered into surveillance (no surveillance group) or having been entered into surveillance. Within the group who were enrolled into surveillance, they were then considered as having been (1) adherent with surveillance or (2) not-adherent with surveillance.

### 2.5. Lead-Time Bias Estimation

Surveillance aims to detect lesions at a pre-symptomatic stage. Patients who have lesions detected by surveillance, therefore, have the additional survival time from when lesions are detected to when they become symptomatic and thus, will appear to have better survival (known as lead-time bias). Three separate approaches were used to account for the effects of lead-time bias on overall survival using:

#### 2.5.1. Rate of Transition to Symptomatic Disease

This approach estimates the additional follow-up time observed purely as a result of lead-time in patients with a surveillance-detected cancer. Described by Duffy et al. [22], the rate of transition is from when a cancer is asymptomatic, but surveillance detectable to a cancer with symptoms is λ. Thus, the expected additional time, *E*(*s*), due to lead-time bias is given by:

(1) $$E\left(s\right)=\;\frac{1-e^{-\lambda t}}{\lambda }$$

where *t* is equal to the survival time from HCC diagnosis observed in a patient with a surveillance-detected cancer. We used data from studies that estimated the time taken for screen-detectable HCC to transition to symptomatic disease to be between 70 to 140 days [23,24]. We also tested this for the time specified as being non-adherent to surveillance (9 months) and for the most extreme values, we could find them in the literature for cirrhotic disease (1.57 years) and non-cirrhotic disease as the upper bound to provide a conservative estimate (2.66 years) [25].

#### 2.5.2. Describing Lead-Time Using Tumour Size

Assuming surveillance detects tumours when they are smaller and asymptomatic, we used the maximum tumour diameter at HCC diagnosis, measured on cross-sectional imaging (either CT or MRI), to generate a scaled and centred variable describing lead-time. We then used a gamma distribution model to predict the scaled lead-time estimation for patients who were not in surveillance, based on the maximum size of their tumours and their BCLC stage (to account for disease spread in addition to just size alone). The gamma distribution was used as lead-times are always positive and have skewed distributions. This variable was then included in models as an interaction term with surveillance group, as the causal effect of surveillance is dependent on lead-time (i.e., patients with early asymptomatic lesions have much longer survival times, as a combination of long lead-times and being in surveillance). We performed this analysis in the Edinburgh cohort, as tumour size was only collected in this cohort.

#### 2.5.3. Counterfactual Estimation

Given there are situations in the management of HCC where there is no anticancer therapy provided and no effective therapy for cirrhosis, we hypothesised that if we observed improved survival in the supportive care only group, the difference would be due to lead-time, rather than due to a survival benefit gained by being in surveillance. We compared the groups who received supportive care only and using a flexible parametric survival model, applied the differences to our study population. We term here the additional survival time for patients with a surveillance-detected cancer *E(scf).* Using these three approaches, we estimated plausible values for the effect of HCC surveillance on overall survival from the point of HCC diagnosis and the effect of surveillance adherence within these patient populations.

### 2.6. Data Handling and Statistical Analysis

Data were summarised using percentages and counts for categorical data, mean average where continuous data were normally distributed (presented with standard deviation—SD) and median average where continuous data did not fit the normal distribution (presented with 25th and 75th centiles). To test for baseline differences across summarised groups, we used the chi-square test (or Fisher’s exact where appropriate) for categorical data, Welch’s t-test for continuous normally distributed data, Wilcoxon signed rank test for non-normal continuous data, or the Kruskal–Wallis test for continuous data where there were more than two groups. To see whether we could identify predictors of entering and adhering to surveillance, multilevel regression modelling was used to account for variation observed across the centres and patient-level characteristics. Patient-level characteristics were modelled as fixed effects, with centre specified as a random effect. For these models, variables were selected on the basis they were clinically relevant and were unlikely to be confounded. Continuous variables were centred or separated into clinically relevant categories prior to inclusion in models. Final model selection was guided by the minimisation of the Akaike information criterion (AIC). Effects estimates are represented as odds ratios (OR) with the corresponding 95% confidence interval (95% CI).

Where missing data for predictor variables were present, multiple imputation by chained equations was planned to impute these data. Missing data rates were very low, and imputation was not required.

For survival analyses, as we were modifying the survival times based on our lead-time bias estimates, we used a flexible parametric model to estimate hazards at a given time. We specified centre as a random effect variable within this model to account for centre-level variation. Survival effect estimates are represented as Hazard Ratios (HR) with the corresponding 95% confidence interval. First order interactions were examined for each variable entered into every multivariable model and significant interactions retained.

Statistical significance was set at the level of *p* < 0.05. All statistical analysis was performed using R version 3.6.3 (R Foundation for Statistical Computing, Vienna, Austria) using the Tidyverse, flexsurv and finalfit packages.

## 3. Results

We included 985 patients diagnosed with HCC in the two centres between 1 January 2009 and 31 December 2015 inclusive. From the Scottish Cancer Registry, for the population covered by the two centres, there were a total number of 1117 incident cases over the same study time period, giving a case ascertainment rate of 88.18%. Of these 985 patients, 638 (64.8%) were from Centre 1 (Glasgow) and 347 (35.2%) were from Centre 2 (Edinburgh). Data are shown by centre in Supplementary Table S1).

Missing data in all fields were ≤1%; therefore, no imputation was required. During the study period between 2009 and 2015, the number of patients diagnosed annually with HCC rose from 96 to 182.

Table 1 shows the characteristics of the included patients; the median age of the patients was 69 years (25th centile—61; and 75th centile—77) and 789 (80.1%) patients were male.

The most common aetiology for underlying liver disease was Alcoholic Liver Disease (47.9%), followed by Non-alcoholic Fatty Liver Disease (23.7%) and viral hepatitis (21.5%; see Table 1). There were no changes in the distribution of patient characteristics over time. Characteristics were similar across centres, with the exception of disease aetiology, where a greater proportion of patients in Centre 1 had ALD (53.0%, 338/638 in Centre 1 versus 38.6%, 134/347 in Centre 2); and for BCLC stage at HCC diagnosis, where Centre 1 had a lower proportion of patients with BCLC Stage 0/A disease (17.1%, 109/638 in Centre 1 versus 41.2%, 143/347 in Centre 2; Supplementary Table S2).

### 3.1. Enrolment and Adherence to Surveillance

Out of the 985 included patients, 591 (60.0%) had not been enrolled in HCC surveillance, 302 (30.7%) had been enrolled and were adherent with surveillance, and 92 (9.3%) had been enrolled but were not adherent with surveillance. The overall adherence for surveillance was 76.6% (302/394). Patients in the surveillance groups were younger than those not in surveillance, were more likely to have ALD, viral hepatitis or ‘other aetiology’ and had lower Child–Pugh stage at diagnosis of HCC (Table 1). Using multilevel modelling, female sex, younger age, the presence of alcoholic liver disease, viral hepatitis and NAFLD were associated with entry into surveillance (Table 2). We found no clinical characteristics predicted adherence to surveillance (Supplementary Table S3).

### 3.2. Stage at Diagnosis

**Figure 1:**
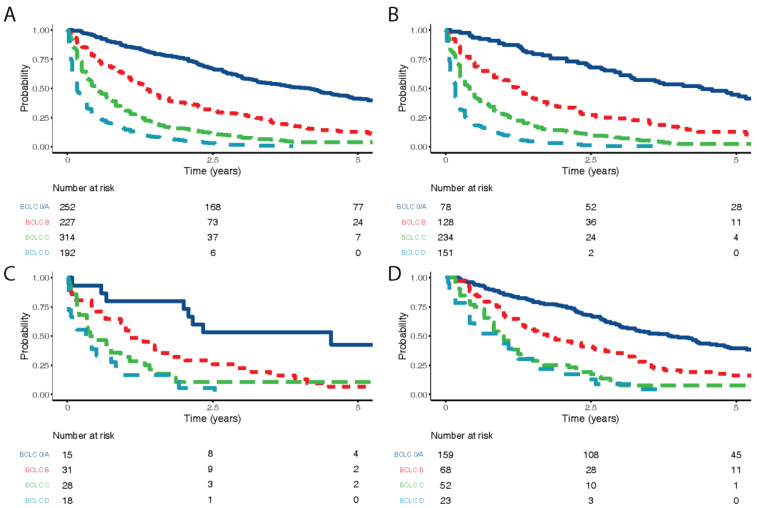
Overall survival by BCLC stage. (**A**) For all patients included in the study, (**B**) for patients never entered into surveillance, (**C**) overall survival by BCLC stage for those not adherent to surveillance, (**D**) overall survival for those adherent with surveillance.

Over half the patients in the adherent with surveillance group (52.6%, 159/302) were diagnosed with BCLC stage 0/A disease, compared to 16.3% (15/92) in the not adherent group and 13.5% (78/591) in the no surveillance group (*p* < 0.001; Table 3). In both the non-adherent to surveillance and not under surveillance groups, disease at presentation was more advanced. Patients with higher BCLC stages had poorer overall survival, which was consistent across all surveillance groups (Figure 1).

### 3.3. Treatment Intent

Patients adherent to surveillance were most likely to receive potentially curative treatments (37%), compared with those not adherent (17.4%) and those not in surveillance (10.8%) *p* < 0.001 (Table 3). When adjusted for potential confounders using a multilevel logistic regression model (Table 4), surveillance remained significantly associated with receipt of curative therapy. When BCLC stage was included as an interaction term due to its impact on receipt of curative therapy, adherence to surveillance remained significantly associated with the use of curative therapies, regardless of BCLC stage (Table 4).

### 3.4. Survival

**Figure 2:**
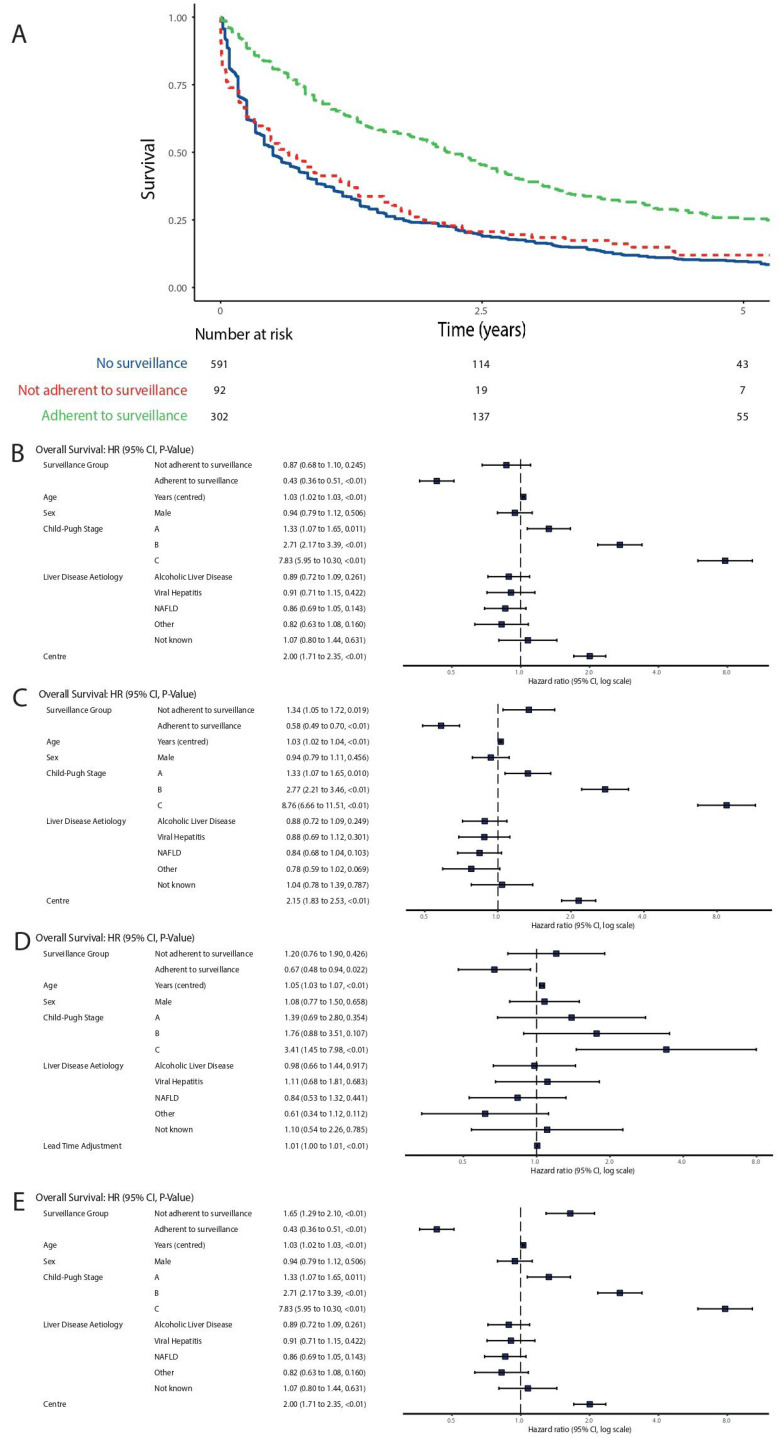
Corrections for lead-time bias. (**A**) Overall survival by surveillance adherence, without adjustment for lead-time, (**B**) flexible parametric survival model for overall survival with no adjustment for lead-time bias, (**C**) flexible parametric survival model for overall survival, adjusted for lead-time bias using rate of transition to symptomatic disease method (270 days), (**D**) flexible parametric survival model for overall survival, adjusted for lead-time bias using tumour size method, (**E**) flexible parametric survival model for overall survival, adjusted for lead-time bias using counterfactual method.

Median overall survival was 6.0 months in the no surveillance group (25th centile 2.0 months, 75th centile 21.0 months), 10.1 months in the not adherent with surveillance group (25th centile 2.4 months, 75th centile 25.2 months) and 28.7 months in the adherent with surveillance group (25th centile 11.4 months, 75th centile 52.0 months). At the time of data collection, a total of 1881 years of follow-up had been accrued by all patients in the study. When lead-time bias was not accounted for, adherence with surveillance was associated with longer survival (Figure 2A). When adjusted for explanatory variables, this association persisted (Figure 2B).

Child–Pugh grade, increasing age and centre were the only other variables associated with survival, with cirrhosis of any grade associated with worse survival (Figure 2B and Table 4).

When using the transition to symptomatic disease method to account for lead-time bias, adherence to surveillance remained associated with a survival benefit (Figure 2C). As a sensitivity analysis, we entered further permutations of lead-time (Supplementary Figure S1, Kaplan–Meier for symptom transition method. A—70 days, B—140 days, C—270 days, D—1.57 years, E—2.66 years). For all permutations of transition time except 2.66 years (conservative estimate in methodology as above), a significant association between survival and adherence to surveillance remained. Patients who were not adherent to surveillance continued to have a similar survival to those who were not entered into surveillance, up to and including 140 days adjustment. When adjusted lead-time exceeded 140 days, we observed worse survival in the not adherent group, supporting the hypothesis that both lead-time and surveillance group contribute to the total observed effect of surveillance for HCC.

When we described lead-time as a variable in our model based on tumour size at presentation in the Edinburgh cohort, we found the beneficial effect of surveillance adherence on overall survival persisted independent of lead-time (Figure 2D). Patients with shorter lead-times/larger tumours at HCC diagnosis had shorter survival.

Finally, using the counterfactual approach, adherence to surveillance was not significantly associated with improved survival, but did not reverse the effect of surveillance (Figure 2E, Supplementary Figure S2).

### 3.5. Sensitivity Analysis

**Figure 3:**
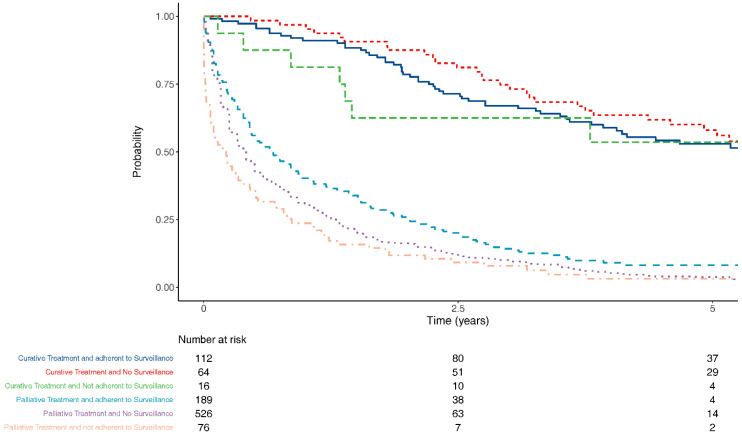
Overall survival by surveillance group and treatment intent, 270 days lead-time bias accounted for.

Patients in surveillance were significantly more likely to receive potentially curative therapies, even when lead-time bias was accounted for (Figure 3).

## 4. Discussion

This study found that in a large patient population undergoing real world surveillance in the two largest Scottish regions, HCC surveillance was associated with earlier disease stage at presentation, increased use of therapy with curative intent, and improved overall survival from diagnosis. However, patients with poor adherence to surveillance had outcomes similar to those never entered into surveillance. The beneficial effect of surveillance on overall survival was lower than that reported in many other studies that did not fully consider lead-time bias.

There is a lack of high-quality, randomised evidence to clarify the best approach to HCC surveillance based either on outcomes or cost efficiency. Most of the evidence regarding the effectiveness of HCC surveillance is from observational studies and only two RCTs have been published, both from China in HBV infected populations [26,27]. One RCT used both USS and AFP for surveillance and reported a 37% reduction in mortality despite suboptimal adherence, whereas the other used AFP measurement only and reported no difference in survival. It is unclear whether the results can be extrapolated to a Western population with different aetiology of liver disease.

Adherence is a critical factor in determining the effectiveness of a given intervention. We found that patients with poor surveillance adherence had similar survival to patients with no surveillance. Indeed, our sensitivity analyses and adjustments for lead-time bias suggested this group may even have poorer survival. This could be an artefactual finding arising from our modelling techniques, or it could be that patients who are poorly adherent may have different behavioural or healthcare-seeking characteristics from the other groups [28]. The importance of adherence to surveillance should be a key component of the initial discussion with a patient who is being considered for entry into an HCC surveillance programme.

Our study has several strengths. Firstly, the study population was highly annotated consecutive patients diagnosed with HCC, with cirrhosis of various aetiologies, and included referrals from smaller local hospitals. Therefore, our study is likely to represent a real-world scenario. Much of the current evidence is from areas in the world where HBV and/or HCV are endemic. It is well described that there are differences in the clinical characteristics and genetic drivers of HCC arising on a background of viral liver disease compared with other aetiologies; therefore, our study helps address this area of uncertainty [29].

We also used a variety of statistical approaches to account for lead-time bias. Most studies of HCC surveillance rely on a single method using assumptions such as tumour volume doubling time, which may be unreliable given the heterogeneous nature of HCC tumours [30]. In addition, by analysis of data from the Scottish Cancer Registry, we confirmed that we managed to capture 88.18% of all HCC cases from our centres over the study period.

Our study has several limitations. The data originate from a prospectively collected observational database. We chose a pragmatic definition of adherence to surveillance defined as a US scan performed within 9 months prior to HCC diagnosis. However, this time period has been used by other studies in this field and takes into account potential organisational delays in coordinating US scans [31,32]. Furthermore, we did not collect detailed data on number of tumours, size of tumour at diagnosis or details on the presence portal hypertension. This was to make the study feasible by minimizing the number of variables to be collected. This meant that we grouped BCLC stage 0 and A together. Although this could be considered an important distinction, patients in both stages are eligible for curative therapy and are likely to have the best prognosis. Therefore, we believe this grouping is unlikely to have had a major effect on our findings.

Given our findings, controversy about the clinical and cost-effectiveness of HCC surveillance is likely to continue. Estimating the true effect of HCC surveillance on overall survival would ideally be carried out in a large RCT. However, this would be extremely challenging due to issues of patient acceptability, controversy around the clinical equipoise of surveillance, current guidelines recommending surveillance, and potential new developments in surveillance technologies. These difficulties were underlined when investigators tested the feasibility of conducting an RCT in 205 patients with liver cirrhosis and 99.5% of patients declined randomisation, with 88% choosing non-randomised surveillance [33]. The fact that only 37.1% patients adhering to surveillance in our study underwent curative therapy underlines the limitations of the surveillance investigations and methods that are currently recommended by most guidelines. This emphasises the importance of finding new and better surveillance tests that may include biomarkers and abbreviated MRI.

In conclusion, current evidence for HCC surveillance in a population with mixed causes of cirrhosis is confounded by lead-time bias. We have demonstrated that the effect size of HCC surveillance on overall survival is smaller than previously described. Despite this, adherence to surveillance is associated with earlier stage at HCC diagnosis, increased access to curative therapies, and increased survival. However, patients with poor adherence to surveillance have outcomes similar to patients never enrolled in surveillance, which is an important educational point to discuss with patients being entered into a surveillance programme.

## Figures and Tables

**Table 1:** Patient characteristics by surveillance adherence group.

	Total N	Missing N	Levels	No Surveillance	Not Adherent to Surveillance	Adherent to Surveillance	*p*
Total N (%)				591 (60.0)	92 (9.3)	302 (30.7)	
Age at diagnosis	985	0	Median (25th, 75th centile)	73.0 (64.5 to 79)	67.0 (60.8 to 73)	64.0 (58 to 71)	<0.001 ^†^
Sex	985	0	Female	106 (17.9)	22 (23.9)	68 (22.5)	0.161
			Male	485 (82.1)	70 (76.1)	234 (77.5)	
Year of diagnosis	985	0	Median (25th, 75th centile)	2012.0 (2011 to 2014)	2012.0 (2011 to 2014)	2013.0 (2011 to 2014)	0.007 ^†^
Child–Pugh Stage at diagnosis	985	0	No Cirrhosis	142 (24.0)	5 (5.4)	10 (3.3)	<0.001
			A	169 (28.6)	32 (34.8)	150 (49.7)	0.023
			B	209 (35.4)	46 (50.0)	114 (37.7)	
			C	71 (12.0)	9 (9.8)	28 (9.3)	
Alcoholic Liver Disease	985	0	No	331 (56.0)	40 (43.5)	142 (47.0)	0.009
			Yes	260 (44.0)	52 (56.5)	160 (53.0)	
Viral hepatitis	985	0	No	513 (86.8)	68 (73.9)	194 (64.2)	<0.001
			Yes	78 (13.2)	24 (26.1)	108 (35.8)	
Non-alcoholic fatty liver disease	985	0	No	447 (75.6)	69 (75.0)	236 (78.1)	0.670
			Yes	144 (24.4)	23 (25.0)	66 (21.9)	
Other	985	0	No	387 (65.5)	69 (75.0)	250 (82.8)	<0.001
			Yes	204 (34.5)	23 (25.0)	52 (17.2)	

All tests are chi-square, except when denoted by † where Kruskal–Wallis tests used. SD—Standard Deviation.

**Table 2:** Model for surveillance group membership (entry to surveillance).

Dependent: Entry to Surveillance		No Surveillance	Entered into Surveillance	OR (Univariable)	OR (Multilevel)
Age at diagnosis	Mean (SD)	70.9 (11.4)	64.8 (9.7)	0.95 (0.94–0.96, *p* < 0.001)	0.97 (0.95–0.98, *p* < 0.001)
Sex	Female	106 (54.1)	90 (45.9)	-	-
	Male	485 (61.5)	304 (38.5)	0.74 (0.54–1.01, *p* = 0.059)	0.60 (0.42–0.85, *p* = 0.004)
Alcoholic Liver Disease	No	331 (64.5)	182 (35.5)	-	-
	Yes	260 (55.1)	212 (44.9)	1.48 (1.15–1.92, *p* = 0.003)	1.75 (1.20–2.55, *p* = 0.004)
Viral hepatitis	No	513 (66.2)	262 (33.8)	-	-
	Yes	78 (37.1)	132 (62.9)	3.31 (2.42–4.56, *p* < 0.001)	2.35 (1.49–3.69, *p* < 0.001)
Non-alcoholic fatty liver disease	No	447 (59.4)	305 (40.6)	-	-
	Yes	144 (61.8)	89 (38.2)	0.91 (0.67–1.22, *p* = 0.520)	1.29 (0.85–1.94, *p* = 0.231)
Other	No	387 (54.8)	319 (45.2)	-	-
	Yes	204 (73.1)	75 (26.9)	0.45 (0.33–0.60, *p* < 0.001)	0.99 (0.62–1.57, *p* = 0.963)

SD—Standard Deviation, OR—Odds Ratio.

**Table 3:** Stage of disease and subsequent therapy by surveillance adherence group.

Label	Total N	Missing N	Levels	No Surveillance	Not Adherent to Surveillance	Adherent to Surveillance	*p*
Total N (%)				591 (60.0)	92 (9.3)	302 (30.7)	
BCLC Stage at diagnosis	985	0	0/A	78 (13.2)	15 (16.3)	159 (52.6)	<0.001
			B	128 (21.7)	31 (33.7)	68 (22.5)	
			C	234 (39.6)	28 (30.4)	52 (17.2)	
			D	151 (25.5)	18 (19.6)	23 (7.6)	
AFP level	941	44	<100	299 (50.6)	61 (66.3)	241 (79.8)	<0.001
			>1000	165 (27.9)	16 (17.4)	16 (5.3)	
			100–1000	87 (14.7)	10 (10.9)	41 (13.6)	
			Not known	5 (0.8)	0 (0.0)	0 (0.0)	
			(Missing)	35 (5.9)	5 (5.4)	4 (1.3)	
Treatment	983	2	Liver resection	36 (6.1)	4 (4.3)	24 (7.9)	<0.001
			Liver transplant	8 (1.4)	3 (3.3)	45 (14.9)	
			Ablative therapies	20 (3.4)	9 (9.8)	43 (14.2)	
			Sorafenib	22 (3.7)	2 (2.2)	7 (2.3)	
			Supportive care only	382 (64.6)	47 (51.1)	85 (28.1)	
			TACE	122 (20.6)	27 (29.3)	97 (32.1)	
			(Missing)	1 (0.2)	0 (0.0)	1 (0.3)	
Treatment type	983	2	Curative therapy	64 (10.8)	16 (17.4)	112 (37.1)	<0.001
			Palliative therapy	144 (24.4)	29 (31.5)	104 (34.4)	
			Supportive care only	382 (64.6)	47 (51.1)	85 (28.1)	
			(Missing)	1 (0.2)	0 (0.0)	1 (0.3)	
Treatment intent	983	2	Curative	64 (10.8)	16 (17.4)	112 (37.1)	<0.001
			Palliative	526 (89.0)	76 (82.6)	189 (62.6)	
			(Missing)	1 (0.2)	0 (0.0)	1 (0.3)	

AFP—Alpha-fetoprotein, BCLC—Barcelona Clinic Liver Cancer, TACE—Transarterial Chemoembolisation. All tests are chi-square.

**Table 4:** Model for receiving potentially curative therapy.

Dependent: Treatment Intent		Palliative	Curative	OR (Univariable)	OR (Multilevel)
Surveillance group	No surveillance/not compliant	602 (88.3)	80 (11.7)	-	-
	Adherent to surveillance	189 (62.8)	112 (37.2)	0.73 (0.43–1.24, *p* = 0.249)	0.67 (0.38–1.20, *p* = 0.182)
BCLC Stage at diagnosis	0/A	98 (39.0)	153 (61.0)	-	-
	B	210 (92.9)	16 (7.1)	0.02 (0.01–0.05, *p* < 0.001)	0.02 (0.01–0.06, *p* < 0.001)
	C	296 (94.3)	18 (5.7)	0.02 (0.01–0.05, *p* < 0.001)	0.02 (0.01–0.05, *p* < 0.001)
	D	187 (97.4)	5 (2.6)	0.00 (0.00–0.01, *p* < 0.001)	0.00 (0.00–0.02, *p* < 0.001)
Age at diagnosis	Median (25th to 75th centile)	71 (63 to 78)	71 (63 to 78)	0.95 (0.93–0.96, *p* < 0.001)	0.95 (0.92–0.97, *p* < 0.001)
Sex	Female	153 (78.1)	43 (21.9)	-	-
	Male	638 (81.1)	149 (18.9)	0.83 (0.57–1.23, *p* = 0.343)	1.06 (0.62–1.80, *p* = 0.829)
Alcoholic Liver Disease	No	406 (79.3)	106 (20.7)	-	-
	Yes	385 (81.7)	86 (18.3)	0.86 (0.62–1.17, *p* = 0.334)	0.69 (0.40–1.18, *p* = 0.173)
Child–Pugh Stage	No Cirrhosis	130 (83.3)	26 (16.7)	-	-
	A	243 (69.4)	107 (30.6)	2.20 (1.38–3.61, *p* = 0.001)	0.66 (0.31–1.37, *p* = 0.264)
	B	319 (86.4)	50 (13.6)	0.78 (0.47–1.33, *p* = 0.354)	0.42 (0.19–0.92, *p* = 0.029)
	C	99 (91.7)	9 (8.3)	0.45 (0.19–0.98, *p* = 0.054)	0.78 (0.19–3.31, *p* = 0.739)
Viral hepatitis	No	648 (83.8)	125 (16.2)	-	-
	Yes	143 (68.1)	67 (31.9)	2.43 (1.71–3.43, *p* < 0.001)	0.96 (0.50–1.88, *p* = 0.913)
Non-alcoholic fatty liver disease	No	608 (81.1)	142 (18.9)	-	-
	Yes	183 (78.5)	50 (21.5)	1.17 (0.81–1.67, *p* = 0.396)	1.41 (0.75–2.64, *p* = 0.288)
Other	No	556 (78.9)	149 (21.1)	-	-
	Yes	235 (84.5)	43 (15.5)	0.68 (0.47–0.98, *p* = 0.044)	1.31 (0.64–2.67, *p* = 0.459)
Adherent to surveillance: BCLC B	Interaction	-	-	4.50 (1.41–14.81, *p* = 0.011)	4.63 (1.41–15.23, *p* = 0.012)
Adherent to surveillance: BCLC C	Interaction	-	-	4.85 (1.51–14.90, *p* = 0.006)	4.46 (1.35–14.72, *p* = 0.014)
Adherent to surveillance: BCLC D	Interaction	-	-	48.37 (6.26–1006.51, *p* = 0.001)	47.55 (4.40–513.48, *p* = 0.001)

BCLC—Barcelona Clinic Liver Cancer, SD—Standard Deviation, OR —Odds Ratio. Curative therapies are Surgery and Transplant.

## Data Availability

Data sharing not applicable.

## Supplementary Materials

The following are available online at https://www.mdpi.com/article/10.3390/jcm10132770/s1, Figure S1: Kaplan–Meier for symptom transition method. A—70 days, B—140 days, C—270 days, D—1.57 years, E—2.66 years, Figure S2: Kaplan–Meier plot by surveillance adherence, with adjustment for counterfactual method, Table S1: Characteristics by Centre, Table S2: Stage of disease by centre and surveillance adherence, Table S3: Predictors of adherence to surveillance (including centre).
