# Supplementary material for: Effect of Hepatocellular Carcinoma Surveillance Programmes on Overall Survival in a Mixed Cirrhotic UK Population: A Prospective, Longitudinal Cohort Study
Source: J Clin Med. 2021 Jun 24;10(13):2770. doi: 10.3390/jcm10132770 (PMC8269358; doi:10.3390/jcm10132770)
Supplement: Supplementary file 1 [file jcm-10-02770-s001.zip › jcm-1230450-supp.pdf]

Supplementary File

Table S1. – Characteristics by Centre.

	Total N	Missing N	levels	Adherent to surveillance - Edinburgh	Adherent to surveillance - Glasgow	No surveillance - Edinburgh	No surveillance - Glasgow	Not adherent to surveillance - Edinburgh	Not adherent to surveillance - Glasgow	*p*-value
Total N (%)				142 (14.4)	160 (16.2)	167 (17.0)	424 (43.0)	38 (3.9)	54 (5.5)	
Age at diagnosis	985 (100.0)	0	Mean (SD)	64.5 (8.4)	64.0 (10.7)	70.4 (12.9)	71.0 (10.7)	65.2 (9.4)	67.6 (9.5)	<0.001
Sex	985 (100.0)	0	Female	38 (26.8)	30 (18.8)	32 (19.2)	74 (17.5)	13 (34.2)	9 (16.7)	0.047
			Male	104 (73.2)	130 (81.2)	135 (80.8)	350 (82.5)	25 (65.8)	45 (83.3)	
Year of diagnosis	985 (100.0)	0	Mean (SD)	2012.3 (1.9)	2013.0 (1.9)	2012.1 (1.7)	2012.3 (2.0)	2011.0 (1.5)	2013.5 (1.4)	<0.001
Child Pugh Stage	985 (100.0)	0	No Cirrhosis	0 (0.0)	10 (6.2)	16 (9.6)	126 (29.7)	0 (0.0)	5 (9.3)	<0.001
			A	67 (47.2)	83 (51.9)	69 (41.3)	100 (23.6)	15 (39.5)	17 (31.5)	
			B	64 (45.1)	50 (31.2)	72 (43.1)	137 (32.3)	22 (57.9)	24 (44.4)	
			C	11 (7.7)	17 (10.6)	10 (6.0)	61 (14.4)	1 (2.6)	8 (14.8)	
Alcoholic Liver Disease	985 (100.0)	0	No	75 (52.8)	67 (41.9)	118 (70.7)	213 (50.2)	20 (52.6)	20 (37.0)	<0.001
			Yes	67 (47.2)	93 (58.1)	49 (29.3)	211 (49.8)	18 (47.4)	34 (63.0)	
Viral hepatitis	985 (100.0)	0	No	88 (62.0)	106 (66.2)	143 (85.6)	370 (87.3)	25 (65.8)	43 (79.6)	<0.001
			Yes	54 (38.0)	54 (33.8)	24 (14.4)	54 (12.7)	13 (34.2)	11 (20.4)	
Non-alcoholic fatty liver disease	985 (100.0)	0	No	111 (78.2)	125 (78.1)	83 (49.7)	364 (85.8)	28 (73.7)	41 (75.9)	<0.001
			Yes	31 (21.8)	35 (21.9)	84 (50.3)	60 (14.2)	10 (26.3)	13 (24.1)	
Other	985 (100.0)	0	No	120 (84.5)	130 (81.2)	137 (82.0)	250 (59.0)	32 (84.2)	37 (68.5)	<0.001
			Yes	22 (15.5)	30 (18.8)	30 (18.0)	174 (41.0)	6 (15.8)	17 (31.5)	

All tests are Chi-square, except when denoted by † where Kruskall-Wallis tests used. SD – Standard Deviation.

Table S2. – Stage of disease by centre and surveillance adherence.

	Total N	Missing N		Adherent to surveillance - Edinburgh	Adherent to surveillance - Glasgow	No surveillance - Edinburgh	No surveillance - Glasgow	Not adherent to surveillance - Edinburgh	Not adherent to surveillance - Glasgow	*p*-value
Total N (%)				142 (14.4)	160 (16.2)	167 (17.0)	424 (43.0)	38 (3.9)	54 (5.5)	
BCLC Stage at diagnosis	985	0	0/A	98 (69.0)	61 (38.1)	39 (23.4)	39 (9.2)	6 (15.8)	9 (16.7)	<0.001
			B	35 (24.6)	33 (20.6)	32 (19.2)	96 (22.6)	16 (42.1)	15 (27.8)	
			C	7 (4.9)	45 (28.1)	60 (35.9)	174 (41.0)	11 (28.9)	17 (31.5)	
			D	2 (1.4)	21 (13.1)	36 (21.6)	115 (27.1)	5 (13.2)	13 (24.1)	
AFP level	941	44	<100	117 (82.4)	124 (77.5)	73 (43.7)	226 (53.3)	21 (55.3)	40 (74.1)	<0.001
			>1000	4 (2.8)	12 (7.5)	34 (20.4)	131 (30.9)	8 (21.1)	8 (14.8)	
			100–1000	17 (12.0)	24 (15.0)	25 (15.0)	62 (14.6)	4 (10.5)	6 (11.1)	
			Not known	0 (0.0)	0 (0.0)	0 (0.0)	5 (1.2)	0 (0.0)	0 (0.0)	
			(Missing)	4 (2.8)	0 (0.0)	35 (21.0)	0 (0.0)	5 (13.2)	0 (0.0)	
Treatment	983	2	Liver resection	7 (4.9)	17 (10.6)	23 (13.8)	13 (3.1)	1 (2.6)	3 (5.6)	<0.001

	Total N	Missing N		Adherent to surveillance - Edinburgh	Adherent to surveillance - Glasgow	No surveillance - Edinburgh	No surveillance - Glasgow	Not adherent to surveillance - Edinburgh	Not adherent to surveillance - Glasgow	*p*-value
			Liver transplant	31 (21.8)	14 (8.8)	1 (0.6)	7 (1.7)	3 (7.9)	0 (0.0)	
			Ablative therapies	22 (15.5)	21 (13.1)	8 (4.8)	12 (2.8)	4 (10.5)	5 (9.3)	
			Sorafenib	3 (2.1)	4 (2.5)	4 (2.4)	18 (4.2)	0 (0.0)	2 (3.7)	
			Supportive care only	31 (21.8)	54 (33.8)	91 (54.5)	291 (68.6)	16 (42.1)	31 (57.4)	
			TACE	47 (33.1)	50 (31.2)	39 (23.4)	83 (19.6)	14 (36.8)	13 (24.1)	
			(Missing)	1 (0.7)	0 (0.0)	1 (0.6)	0 (0.0)	0 (0.0)	0 (0.0)	
Treatment type	983	2	Curative therapy	60 (42.3)	52 (32.5)	32 (19.2)	32 (7.5)	8 (21.1)	8 (14.8)	<0.001
			Palliative therapy	50 (35.2)	54 (33.8)	43 (25.7)	101 (23.8)	14 (36.8)	15 (27.8)	
			Supportive care only	31 (21.8)	54 (33.8)	91 (54.5)	291 (68.6)	16 (42.1)	31 (57.4)	
			(Missing)	1 (0.7)	0 (0.0)	1 (0.6)	0 (0.0)	0 (0.0)	0 (0.0)	
Treatment intent	983	2	Curative	60 (42.3)	52 (32.5)	32 (19.2)	32 (7.5)	8 (21.1)	8 (14.8)	<0.001
			Palliative	81 (57.0)	108 (67.5)	134 (80.2)	392 (92.5)	30 (78.9)	46 (85.2)	
			(Missing)	1 (0.7)	0 (0.0)	1 (0.6)	0 (0.0)	0 (0.0)	0 (0.0)	

BCLC – Barcelona Clinic Liver Cancer, SD – Standard Deviation, OR – Odds Ratio. Curative therapies are Surgery and Transplant.

Table S3. - Predictors of adherence to surveillance (including centre).

Dependent: Surveillance Adherence		Not adherent to surveillance	Adherent to surveillance	OR (univariable)	OR (multivariable)
Age at diagnosis	Mean (SD)	66.6 (9.5)	64.2 (9.7)	0.97 (0.95-1.00, *p* = 0.042)	0.98 (0.95-1.01, *p* = 0.132)
Sex	Female	22 (24.4)	68 (75.6)	-	-
	Male	70 (23.0)	234 (77.0)	1.08 (0.61-1.85, *p* = 0.780)	1.06 (0.57-1.90, *p* = 0.857)
Alcoholic Liver Disease	No	40 (22.0)	142 (78.0)	-	-
	Yes	52 (24.5)	160 (75.5)	0.87 (0.54-1.38, *p* = 0.551)	0.70 (0.38-1.27, *p* = 0.240)
Viral hepatitis	No	68 (26.0)	194 (74.0)	-	-
	Yes	24 (18.2)	108 (81.8)	1.58 (0.95-2.70, *p* = 0.087)	0.92 (0.45-1.94, *p* = 0.832)
Non-alcoholic fatty liver disease	No	69 (22.6)	236 (77.4)	-	-
	Yes	23 (25.8)	66 (74.2)	0.84 (0.49-1.47, *p* = 0.528)	0.77 (0.40-1.51, *p* = 0.445)
Other	No	69 (21.6)	250 (78.4)	-	-
	Yes	23 (30.7)	52 (69.3)	0.62 (0.36-1.10, *p* = 0.098)	0.57 (0.27-1.18, *p* = 0.123)
Centre	Edinburgh	38 (21.1)	142 (78.9)	-	-
	Glasgow	54 (25.2)	160 (74.8)	0.79 (0.49-1.27, *p* = 0.336)	0.86 (0.52-1.40, *p* = 0.540)

SD – Standard Deviation.

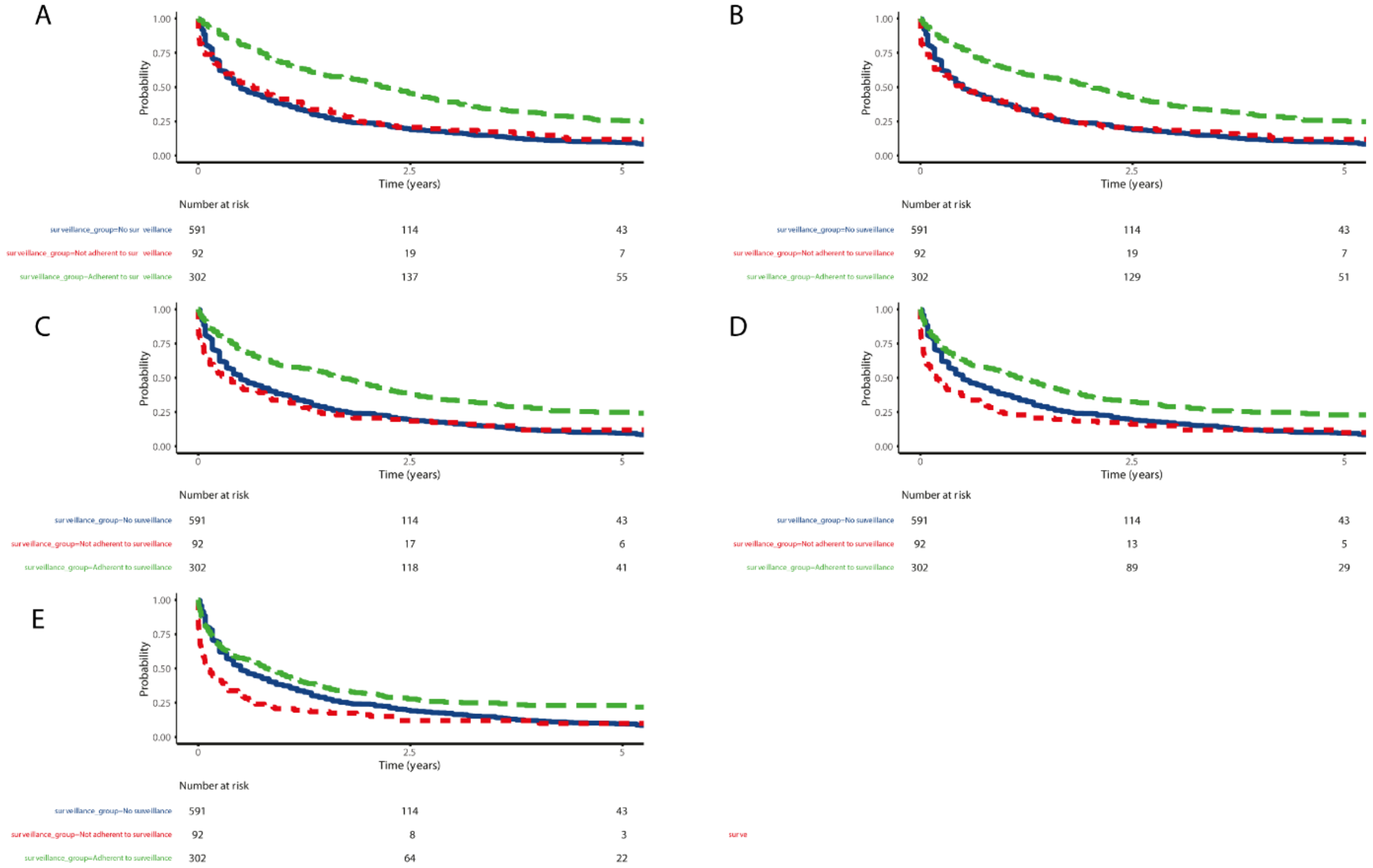

Figure S1. Kaplan-Meier for symptom transition method. **A** – 70 days, **B** – 140 days, **C** – 270 days, **D** – 1.57 years, **E** – 2.66 years.

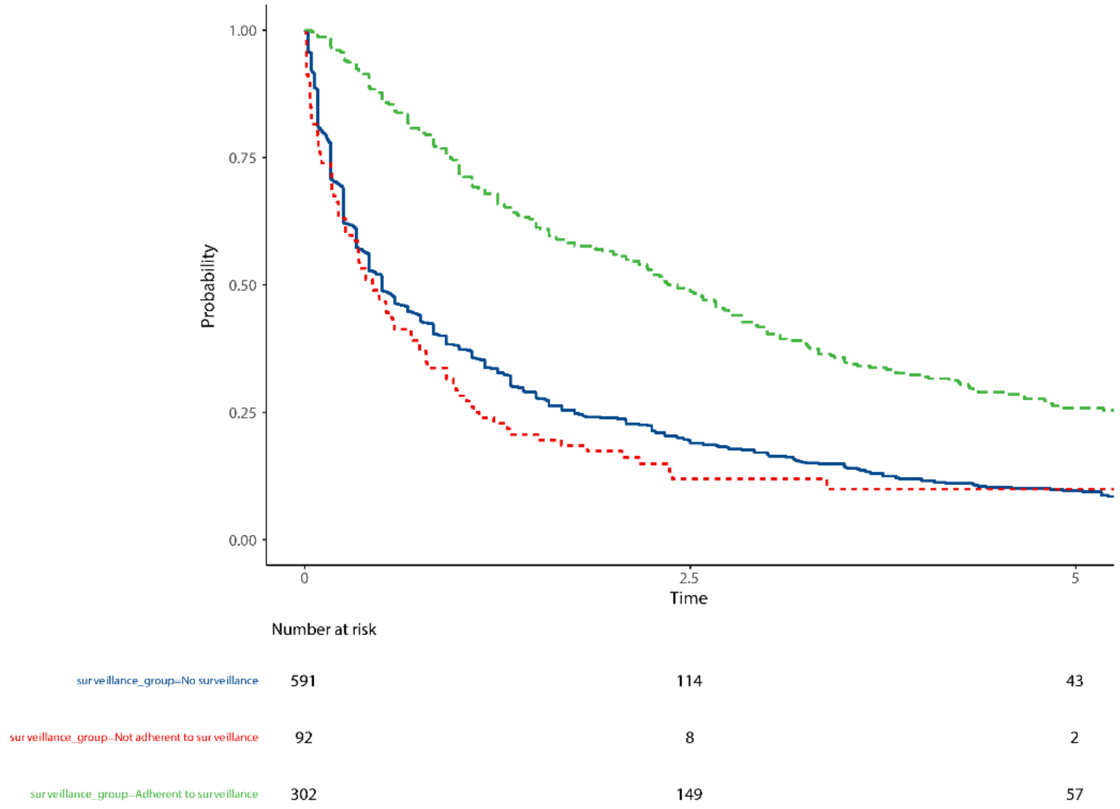

Figure S2. – Kaplan-Meier plot by surveillance adherence, with adjustment for counterfactual method.
